# Elucidating the Role of Optical Activity of Polymers in Protein–Polymer Interactions

**DOI:** 10.3390/polym16010065

**Published:** 2023-12-24

**Authors:** Samin Jahan, Catherine Doyle, Anupama Ghimire, Diego Combita, Jan K. Rainey, Brian D. Wagner, Marya Ahmed

**Affiliations:** 1 Department of Chemistry, University of Prince Edward Island, Charlottetown, PE C1A 4P3, Canada; sjahan14868@upei.ca (S.J.); cdoyle2@upei.ca (C.D.); dcombitamerchan@upei.ca (D.C.); bwagner@upei.ca (B.D.W.); 2 Department of Biochemistry & Molecular Biology, Dalhousie University, Halifax, NS B3H 4R2, Canada; an696021@dal.ca (A.G.); jan.rainey@dal.ca (J.K.R.); 3 Department of Chemistry, Dalhousie University, Halifax, NS B3H 4R2, Canada; 4 School of Biomedical Engineering, Dalhousie University, Halifax, NS B3H 4R2, Canada; 5 Faculty of Sustainable Design Engineering, University of Prince Edward Island, Charlottetown, PE C1A 4P3, Canada

**Keywords:** chiral materials, protein–polymer interactions, antifouling, protein stabilizing

## Abstract

Proteins are biomolecules with potential applications in agriculture, food sciences, pharmaceutics, biotechnology, and drug delivery. Interactions of hydrophilic and biocompatible polymers with proteins may impart proteolytic stability, improving the therapeutic effects of biomolecules and also acting as excipients for the prolonged storage of proteins under harsh conditions. The interactions of hydrophilic and stealth polymers such as poly(ethylene glycol), poly(trehalose), and zwitterionic polymers with various proteins are well studied. This study evaluates the molecular interactions of hydrophilic and optically active poly(vitamin B5 analogous methacrylamide) (poly(B5AMA)) with model proteins by fluorescence spectroscopy, nuclear magnetic resonance (NMR) spectroscopy, and circular dichroism (CD) spectroscopy analysis. The optically active hydrophilic polymers prepared using chiral monomers of *R*-(+)- and *S*-(−)-B5AMA by the photo-iniferter reversible addition fragmentation chain transfer (RAFT) polymerization showed concentration-dependent weak interactions of the polymers with bovine serum albumin and lysozyme proteins. Poly(B5AMA) also exhibited a concentration-dependent protein stabilizing effect at elevated temperatures, and no effect of the stereoisomers of polymers on protein thermal stability was observed. NMR analysis, however, showed poly(B5AMA) stereoisomer-dependent changes in the secondary structure of proteins.

## 1. Introduction

Proteins are biomaterials with specific target applications in various fields, including pharmacology, food sciences, the agriculture industry, biotechnology, and drug delivery [1,2]. Proteins, however, are unstable and prone to aggregation when exposed to external stressors such as heat, agitation, pH changes, and desiccation that increase costs during production and storage and limit their applications in the food industry and as biopharmaceutics [1,2,3,4,5,6]. The physical and chemical interactions of proteins with inert polymers such as polyethylene glycol (PEG), poly(sulfobetaine methacrylate) (p(SBMA)), and poly(carboxybetaine methacrylate) (p(CBMA)) is a well-studied approach to improve the biocompatibility and therapeutics efficacies of protein-based drugs [3,4,5,6,7,8]. The conjugation of polymers with therapeutic proteins improves blood circulation time by reducing interactions with blood plasma, imparting proteolytic resistance, and preventing the opsonization of therapeutic proteins in vivo [1,2,7,8,9,10,11,12,13]. In addition, the incorporation of inert polymers such as PEG and poly(trehalose) as excipients improves the stability of proteins under physical stress such as heat, agitation, and desiccation [5,6].

PEG, p(SBMA), and p(CBMA) are also documented for their antifouling behaviour, and polymer-chain-tethered surfaces exhibit reduced protein fouling on the modified surface [14,15,16]. Studies on the molecular interactions of stealth polymers with proteins in solution, however, show that stealth polymers such as PEG weakly interact with the proteins, and protein–polymer interactions are largely dependent on the size, concentration, and end group functionalities of PEG chains [17,18,19,20,21,22,23,24,25]. Dynamic, weak, and reversible physical interactions between PEG and proteins are mainly driven by hydrogen bonding and hydrophobic interactions that alter the hydration shell of proteins, hence providing conformational stability to the biomolecules [17,18,19,20,21,22,23,24,25]. Similarly, poly(trehalose) is documented to improve stability by forming hydrogen bonding with proteins and acting as a ‘hydration shell’ that maintains the tertiary structure of the protein in solution [6]. Recently, regioisomers of poly(trehalose) were developed by a free radical polymerization approach and used as excipients to stabilize insulin at elevated temperatures and under agitation conditions [6]. In contrast, a zwitterionic, hydrophilic polymer such as p(SBMA) showed negligible interactions with lysozyme (LYZ) and bovine serum albumin (BSA), as was determined by fluorescence spectroscopy [26,27]. These results indicate that the physicochemical properties of hydrophilic and stealth polymers are largely dictated by their monomeric units and must be evaluated in detail for their interactions with proteins at the molecular level.

We have recently reported the synthesis of biomimetic polymers of vitamin B5 analogous methacrylamide (B5AMA) through a living radical polymerization approach and have shown that the poly(B5AMA) produced is highly hydrophilic and exhibits antifouling properties when grafted on a solid support [28,29,30,31]. Owing to the strong hydration tendency, poly(B5AMA) of different molecular weights exhibited the depletion aggregation of bacteria [29], and surface-tethered poly(B5AMA) chains showed low fouling behaviour for both proteins and bacteria [28]. Furthermore, the polymerization of chiral *R*-(+)- and *S*-(−)-B5AMA monomers by photo-iniferter RAFT polymerization was recently reported to yield optically active polymers with complementary mirror-image cotton effects on each other [30]. Despite the known role of poly(B5AMA) as an antifouling layer when grafted on a glass surface, the solution properties of stereoisomers of poly(B5AMA) and their interactions with proteins in solution remain unknown.

The main focus of this study is to evaluate the effect of the nonspecific physical interactions between hydrophilic and optically active poly(*R*-(+)-B5AMA), poly(*S*-(+)-B5AMA), and racemic poly(*R*/*S*-(+/−)-B5AMA) and model proteins BSA and LYZ in solution, in comparison with PEG of a similar molecular weight. Optically active poly(*R*-(+)-B5AMA)_38_, poly(*S*-(+)-B5AMA)_38_, and poly(*R*/*S*-(+/−)-B5AMA)_38_ of 10 kDa were prepared by the photo-iniferter RAFT polymerization approach and the potential for intermolecular interactions with model proteins was tested in solution by intrinsic tryptophan (Trp) fluorescence spectroscopy and 8-anilino-1-naphthalenesulfonic acid (ANS) fluorescence assay. The changes in the secondary structure of proteins, as a function of the concentration of optically active stereoisomers of poly(B5AMA), were compared with PEG of a similar molecular weight and were analyzed by circular dichroism (CD) spectroscopy and ^1^H-NMR analysis. The stability of proteins in the presence of hydrophilic polymers was further evaluated by temperature-dependent CD measurements. Our data demonstrate that stereoisomers of poly(B5AMA) can stabilize proteins as a function of polymer concentration at elevated temperatures, as determined by fluorescence spectroscopy, NMR, and CD spectroscopy.

## 2. Material and Methods

### 2.1. Materials

4-[[[(2-carboxyethyl) thio] thioxomethyl] thio]-4-cyano-pentanoic acid (TCT-1, 95%, Sigma-Aldrich, Oakville, ON, Canada), 3-(trimethylsilyl)-1-propanesulfonic acid sodium salt (TMSP, 97%, Sigma-Aldrich, Oakville, Canada), methanol (99.9%, Fisher, Ottawa, Canada), N,N-dimethylformamide (DMF, 99.9%, Sigma-Aldrich, Oakville, ON, Canada), (*R*)-pantolactone (99%, Sigma-Aldrich, Oakville, Canada), (*S*)-pantolactone (97%, Ambeed, IL, USA), deuterium oxide (99%, Cambridge Isotope Laboratories Inc., Quebec, QC, Canada), poly(ethylene glycol) methyl ether) (PEG, MN of 10000, Sigma-Aldrich, Oakville, ON, Canada), bovine serum albumin (BSA, 98%, Sigma-Aldrich, Oakville, Canada), lysozyme (LYZ, Biobasic Canada, Markham, ON, Canada), phosphate-buffered saline tablets (Millipore-Sigma, Burlington, MA, USA), and chloroform-d (99.8%, Sigma-Aldrich, Oakville, ON, Canada) were used as received. Vitamin B5 analogous methacrylamide *R*-(+)- and *S*-(−)-B5AMA monomers were synthesized according to a previously reported procedure [30] and analyzed using ^1^H NMR and ^13^C NMR (Figures S1–S4). 

### 2.2. Photopolymerization of B5AMA

Photopolymerization of *R*-(+)- and *S*-(−)-B5AMA was conducted using a photo-iniferter RAFT technique, according to previously established methods [30]. A total of 10% *v*/*v* methanol in a deionized water solution was used as a solvent, and 4-[[[(2-carboxyethyl) thio] thioxomethyl] thio]-4-cyano-pentanoic acid (TCT-1) was used as a photo-iniferter. Briefly, a 5 mL two-neck flask was charged with *R*-(+)-B5AMA (774 mg, 3 mmol) and TCT-1 (24 mg, 0.08 mmol, target degree of polymerization (Dp) = 38) dissolved in 3 mL of solvent. TMSP was used as an internal standard for the determination of monomer conversion. The reagents solution was degassed by three freeze–vacuum–thaw cycles, the sealed reactor was placed inside the chamber of a photoreactor, and the liquid was stirred using a magnetic stirrer plate. Then, the blue light was turned on to mark the start of the reaction. The reaction samples were irradiated using a commercial blue LED bulb (NOMA LED A19, 4.5 W) (Canadian Tire, Charlottetown, Canada), Samples of the reaction media were collected after 24 h of reaction and diluted with deuterium oxide (D_2_O) for ^1^H NMR analysis. *R*-(+)- and *S*-(−)-B5AMA conversion was determined by comparing the resonance at 5.68 ppm of vinyl peaks of the monomer to the methyl resonance of the internal standard at 0.0 ppm. At the end of the reaction, the polymers were purified by dialysis against deionized water for at least 48 h. The water was eliminated by freeze-drying to recover the solid polymers. The statistical copolymer poly (*R*/*S*-(+/−)-B5AMA)_38_ was prepared by an identical method, with a target DP of 19 for each monomer. 

### 2.3. Optical Activity

D-line-specific optical rotations ([α]^25^_D_) were measured in 1% *w*/*v* polymer solutions in deionized water at 25 °C using an Optical Activity AA-5 polarimeter (Optical Activity Ltd., Cambridgeshire, England). 

### 2.4. Gel Permeation Chromatography (GPC) 

The molecular weights and molecular weight distributions of the polymers were obtained using a conventional Agilent Technologies GPC 1260 Infinity system (Agilent, Mississauga, ON, Canada) equipped with a refractive index (RI) detector and two PolarGel-M columns (8 µm, 7.5 × 300 mm). A LiBr solution in DMF (0.5% *w*/*v*) was used as the mobile phase with a flow rate of 1.0 mL/min and a temperature of 30 °C. A set of poly(2-hydroxyethyl methacrylate) (poly(HEMA)) standards with molecular weights ranging from 2.11 kDa to 88.8 kDa (dn/dc 0.145–1.52) (Scientific Polymer Products) were used for calibration. Relative molecular weights of the stereoismers poly(B5AMA), namely, poly(*R*-(+)-(B5AMA)_38,_ poly(*S*-(+)-(B5AMA)_38_, and poly(*R*/*S*-(+/−)-B5AMA)_38_, were obtained using the calibration curve prepared with poly(HEMA) standards.

### 2.5. Fluorescence Spectroscopy

Steady-state fluoroscopy studies were conducted utilizing a VarioskanTM LUX multi-technology microplate reader (Thermo Scientific, Waltham, MA, USA). Fluorescence intensity was recorded upon adding different mass ratios of polymers to proteins as follows: 0, 0.5, 1, 2, 4, 8. The concentration of protein solution remained constant at 1mg/mL in PBS with a pH of 7.4. Fluorescence emission spectra were recorded between 300 and 400 nm (1 nm step size) upon excitation at 279 nm with an excitation and emission slit bandwidth of 5 nm. The highest signal intensity of each abovementioned mass ratio of polymer to protein was picked and plotted at each time point. 

8-anilino-1-naphthalenesulfonic acid (ANS) is an extrinsic fluorescence dye that exhibits a blue-shifted emission spectrum and significantly increased emission intensity when binding to hydrophobic sites. For example, the intensity of its fluorescence is nearly 200 times larger in ethanol than in an aqueous solution [32]. This large sensitivity of ANS fluorescence to the polarity and nature of its microenvironment is a result of a combination of factors, including the effect of polarity on its excited-ground state energy gap and specific solvent–solute interactions [33]. Hence, ANS fluorescence intensity changes are related to alterations in the conformation of the protein and/or the formation of polymer–protein interactions during the folding and unfolding process of the proteins [25,26]. In this study, emission was recorded between 400 and 600 nm, with an excitation wavelength of 350 nm, and the maximum emission wavelength was conducted and graphed over time. Proteins and ANS were mixed at a 1:100 molar ratio and incubated for 15 min prior to polymer addition. Protein concentrations remained constant (1 mg/mL) while polymer-to-protein mass ratios of 0, 0.5, 1, 2, 4, and 8 were added. All the experiments were conducted at room temperature. 

### 2.6. CD Spectroscopy

To evaluate the potential for change in the BSA secondary structure upon polymer interaction, far-UV CD spectra were collected in triplicate using a DSM20 CD spectrophotometer (Olis, Bogart, Georgia) at room temperature (22 ± 1 °C), with all optical slits set at a bandpass of 5.0 nm. Samples were prepared containing 1:1 (*w*/*w*) mixtures of each polymer: BSA, BSA alone, or each polymer alone in phosphate-buffered saline at pH 7.4 at a final concentration of 1 mg/mL BSA and either 1 mg/mL or 8 mg/mL polymer. Spectra were acquired from 260 to 190 nm with a 0.875 nm step size using quartz cuvettes of a 0.5 mm path length (Hellma Canada Limited; Concord, ON, USA). Spectra were averaged and blank corrected. For polymer-containing mixtures, different spectra were determined by subtraction of the CD spectrum of the corresponding polymer sample from that of the mixture. In each instance, the mean residue ellipticity ([θ]) was calculated for BSA on the basis of absorbance at 280 nm (molar absorptivity of 4.4 × 10^4^ M^−1^cm^−1^) [34].

### 2.7. Thermal Denaturation

Temperature-dependent CD measurements at 222 nm were performed by averaging for 30 s at a given temperature on a DSM20 CD spectrophotometer equipped with a thermoelectric CD 250 temperature-controlled cuvette holder (Olis, Athens, GA, USA) using a quartz cuvette with a 1-mm path length, with all optical slits set at a bandpass of 5.0 nm. Sample temperatures were progressively increased from 10 °C to 90 °C in 5 °C increments, with a 2 min incubation after achieving each temperature setpoint prior to measurement. Samples were prepared as described above, containing 1:1 and 8:1 (*w*/*w*) mixtures of polymer: BSA, BSA alone, or each polymer alone, with ellipticity values evaluated following blank subtraction, smoothing, and normalization. The graph was smoothed using the weighted moving average (WMA) function. Trendlines to the normalized temperature-dependent CD signal at 222 nm were fit in MATLAB (The MathWorks Inc., Natick, MA, USA) using the logistic sigmoidal model as a function of temperature (T):

f(T) = a/(1+ exp(−b × (T − T_m_))) (1)

where a is the horizontal asymptote; b is the growth rate parameter; and T_m_ is the midpoint/inflection point giving the thermal denaturation melting temperature.

### 2.8. NMR Spectroscopy

#### 2.8.1. Polymer Conversion Studies

NMR spectroscopy was carried out on a Bruker Avance NMR spectrometer (Bruker, Billerica, MA, USA) operating at 300 MHz for ^1^H and 75 MHz for ^13^C using CDCl_3_ and D_2_O (Cambridge Isotope Laboratories, Quebec, QC, Canada) as solvents.

#### 2.8.2. Protein Interaction Studies

^1^H NMR experiments were performed using a 400 MHz Bruker Avance III ^1^H-NMR spectrometer spectrometer (Bruker, Billerica, MA, USA). Interactions of the polymers (10 mg/mL) with BSA were determined by chemical shift titration from a series of NMR experiments acquired using solvent suppression. To do this, titration experiments were conducted by varying the polymer:protein mass ratios from 0, 1:1, and 2:1, respectively. The solvent used in all cases was D_2_O and all spectra were acquired at room temperature. 

## 3. Results and Discussion

Optically active poly(B5AMA) of predetermined molecular weights and narrow polydispersity were synthesized using *R*-(+)- and *S*(−)-B5AMA monomers by a photo-iniferter RAFT polymerization approach [30]. The molecular weights of the polymers obtained were analyzed by GPC and the near-monodisperse polymers of ~9 kDa (PDI 1.13–1.14) obtained were evaluated for optical activity (Table 1 and Figure S5). As shown in Table 1, optically active ~9 kDa polymers of B5AMA were complementary mirror images to each other [30] and showed optical activities of +31° and −35° for poly(*R*-(+)-(B5AMA)_38_ and poly(*S*-(+)-(B5AMA)_38_, respectively. Poly(*R*/*S*-(+/−)-B5AMA)_38_ prepared through the polymerization of a racemic mixture of chiral monomers yielded optically inactive polymers [α = −4.8°] of ~9 kDa. PEG of 10 kDa was used as a standard control and, as expected, showed no optical activity. 

PEG is well documented as demonstrating molecular-weight-dependent protein interactions. Namely, PEG chains of above 5 kDa have been shown to weakly interact with proteins and change their microenvironment; however, this effect is concentration-dependent [20,21,22,23,24,25]. Poly(B5AMA) and PEG of similar molecular weights were thus chosen to compare the interactions of polymers with model proteins in solution.

### 3.1. Fluorescence Spectroscopy 

Steady-state fluorescence spectroscopy is a widely utilized, rapid, and proven method of gaining insight into protein–polymer interactions in solution by studying the changes in the molecular environment of chromophore agents [25,26]. LYZ and BSA are two abundant proteins that are typically used as models to study the non-fouling behaviour of the materials. At physiological pH, LYZ is a small, compact, and positively charged (14.4 kDa, pI = 11.3) protein, while BSA is a large, negatively charged (66.4 kDa, pI = 4.7) protein. LYZ and BSA contain Trp and tyrosine (Tyr) residues that can be excited at ~280 nm, and the evaluation of changes in the fluorescence emissions of the chromophores provides a measurable response to the conformational changes of proteins in the local microenvironment [25]. Thus, changes in the fluorescence emission intensity of proteins can be attributed to changes in the conformation of BSA and LYZ upon interactions with the polymers [25,26]. Previous studies demonstrated that the fluorescence associated with the presence of Trp residues in protein solutions depend on both protein concentration and time. As the local environment of the Trp residues changes, the intensity of their fluorescence also changes. In general, the intensity of the fluorescence is higher in an apolar environment, for example, the core of folded proteins. In consequence, changes in fluorescence indicate a conformational change in the protein, i.e., partially unfolded protein molecules, which are in equilibrium with the native state and could promote the assembly of proteins to form aggregates [25,35,36]. 

In this study, BSA (16–2000 µg/mL) was excited at 279 nm (spectral bandwidth fwhm = 5 nm), and the change in fluorescence intensity as a function of concentration was recorded. The emission spectra of the protein, assuming that the emission observed above 330 nm predominantly arose from Trp fluorescence [36], recorded over 300–400 nm showed a slight shift (2–3 nm) in the Trp emission maximum (338–343 nm) as a function of protein concentration (Figure S6). The fluorescence intensity of Trp decreased over time regardless of BSA concentration, suggesting protein transformation into a less compact structure and the exposure of Trp residues to the aqueous environment. As previously documented [25,26], lower concentrations of proteins experience faster changes in protein structure and vice versa, a phenomenon attributed to protein crowding effects that slow down protein unfolding at high concentrations (Figure S7). 

To study the interaction of polymers with proteins, a low protein concentration (32 µg/mL) was prepared by serial dilution from the original 1 mg/mL BSA solution as the larger fluorescence signal change associated with rapid partial protein unfolding at this concentration regime enabled a more straightforward characterization of the unfolding process in response to both polymer type and concentration in the protein environment. BSA and LYZ (at 32 µg/mL) were mixed with hydrophilic polymers (PEG, poly (*R*-(+)-B5AMA_38_), poly (*S*-(−)-B5AMA_38_), and poly (*R*/*S*-(+/−)-B5AMA_38_) at various weight/weight ratios (0–8 *w*/*w*) and steady-state emission spectroscopy was performed to evaluate the changes in protein conformation in the presence and absence of polymers at a range of time points (0–24 h). 

**Figure 1:**
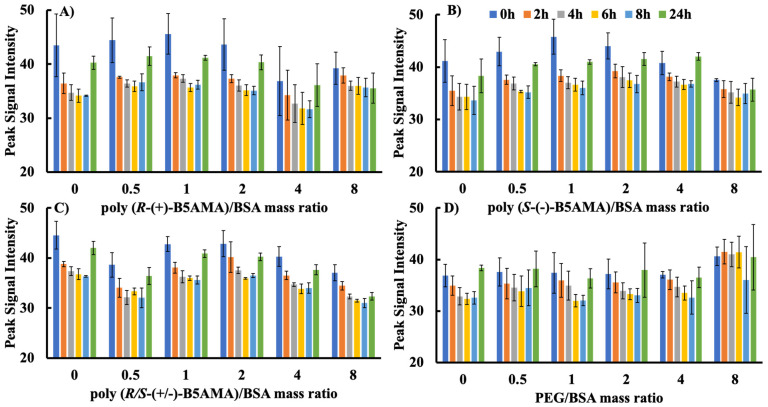
Study of Trp fluorescence intensity of BSA (concentration: 32 µg/mL) upon adding different *w*/*w* ratios (0, 0.5, 1, 2, 4, and 8) of polymer/protein in PBS of pH 7.4 for (**A**) poly(*R*-(+)-B5AMA_38_), (**B**) poly(*S*-(+)-B5AMA_38_), (**C**) poly(*R*/*S*-(+)-B5AMA_38_), and (**D**) PEG at various time points (0, 2, 4, 6, 8, and 24 h). The average values are presented and the error bars represent the standard deviation (SD) for three individual measurements. “0” represents pure BSA solution in the experiments.

The emission maxima of BSA in the presence of all polymers changed slightly (336–343 nm) and the fluorescence signal intensity of proteins alone was relatively similar to that observed at various polymer/protein ratios as a function of time (Figure 1 and Figures S8–S11). As shown in Figure 1, the fluorescence intensity of BSA in the absence of polymers decreased (fluctuating between 45 and 35 AU) in the first 6 h and then increased after the 8 h time point, indicating either the dynamic process of the folding and unfolding of proteins in the aqueous solution or the formation of aggregates, which may have created an apolar environment that increased the Trp fluorescence intensities. The addition of poly(B5AMA) stereoisomers to BSA at the start of the experiment (0 h) showed no meaningful change in overall Trp fluorescence intensity and the values were independent of the type of polymer added to the protein solution. The lack of any change in the fluorescence intensity of proteins in the presence of all polymers at all studied time points and at low polymer/protein *w*/*w* ratios (up to 2) suggests a lack of detectable changes in the protein microenvironment. This is in contrast to other studies, where time-dependent changes in fluorescence intensity have been attributed to interactions between polymers and proteins [25,26]. However, a noticeable decrease in BSA Trp fluorescence intensity was observed at high poly(B5AMA)/protein *w*/*w* ratios (4 and 8) at all studied time points. The reduced fluorescence signal of poly(B5AMA)-BSA systems prepared at high polymer/protein *w*/*w* ratios may be attributed to the changes in the polar environment around the Trp residues. Telechelic polymers prepared by the RAFT polymerization are well documented for their strong absorbance in UV-visible regions, providing unique colours to polymers associated with the functional groups of chain transfer agents (CTAs) present in the polymer structure [37,38]. Indeed, the poly(B5AMA) exhibited strong absorbance in the UV-visible region (270–350 nm; Figure S12). Quenching through, e.g., resonance energy transfer may thus be inferred, leading to the reduced fluorescence intensity observed at higher poly(B5AMA) concentrations. 

In contrast to poly(B5AMA), PEG did not exhibit a concentration-dependent change in the fluorescence intensity of Trp residues of BSA at 0 h. A PEG-concentration-dependent protein stabilization effect was observed over time. This was evidenced by an increase in fluorescence signals compared with protein alone at a *w*/*w* ratio of >4. These observations are consistent with prior studies of BSA–PEG interactions [20,21,22,23,24,25].

**Figure 2:**
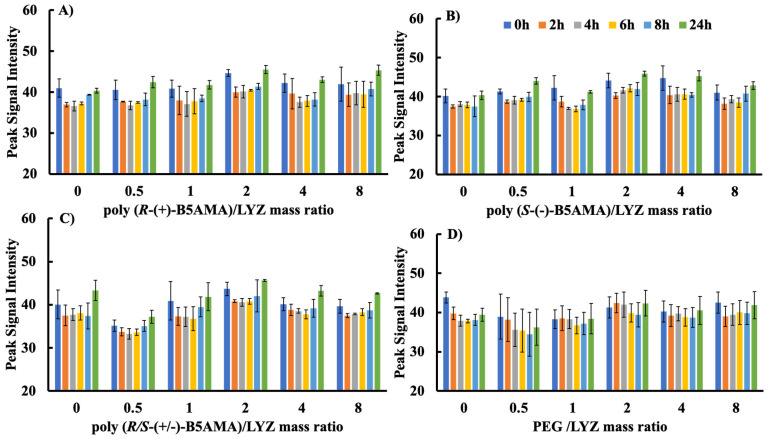
Study of Trp fluorescence intensity of LYZ (concentration: 32 µg/mL) upon adding different mass ratios (0, 0.5, 1, 2, 4, and 8) of polymer/protein in PBS of pH 7.4 for (**A**) poly(*R*-(+)-B5AMA_38_), (**B**) poly(*S*-(+)-B5AMA_38_), (**C**) poly(*R*/*S*-(+)-B5AMA_38_), and (**D**) PEG at various time points (0, 2, 4, 6, 8, and 24 h). The average values are presented and the error bars represent the standard deviation (SD) for three individual measurements. “0” represents pure LYZ solution in the experiments.

To evaluate the changes in the fluorescence intensity of protein–polymer complexes with a small and rigid protein, polymer effects were evaluated using LYZ at low concentrations (32 µg/mL; Figure 2). LYZ alone showed subtle changes in Trp fluorescence intensity compared to BSA, with an overall 5% decrease over a period of 6 h, followed by a slight increase at 8 and 24 h. For all stereoisomers of poly(*R*-B5AMA)_38_-LYZ systems prepared at all polymer/protein mass ratios (0.5, 1, 2, 4, and 8), there was no change in fluorescence intensity compared to LYZ alone for up to 24 h, indicating no obvious changes in the microenvironments of the Trp residues in LYZ due to protein–polymer interactions. A total of 10 kDa PEG used as a control showed polymer-concentration-dependent changes in the Trp fluorescence intensity of LYZ, indicating PEG–LYZ complex formation at a *w*/*w* ratio of >4. This is consistent with previous observations of an increase in fluorescence due to the stabilization of LYZ by the PEG layer [26]. In contrast to BSA, the lack of change in the Trp fluorescence of LYZ for all stereoisomers of poly(B5AMA) is consistent with limited interactions between the CTA of the polymers and Trp residues of the rigid LYZ protein.

**Figure 3:**
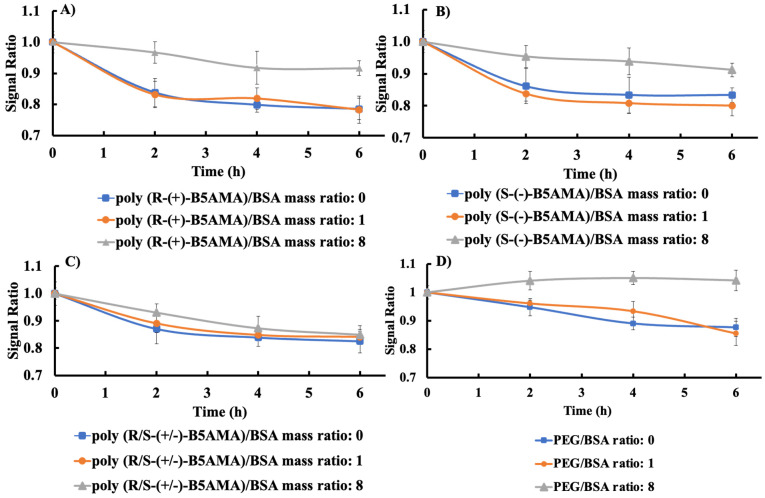
Normalized fluorescence intensity of BSA with and without (**A**) poly(*R*-(+)-B5AMA_38_), (**B**) poly(*S*-(+)-B5AMA_38_), (**C**) poly(*R*/*S*-(+)-B5AMA_38_), and (**D**) PEG. Normalized fluorescence intensity was determined at polymer/protein mass ratios of 1 and 8 at different time points. The average values are presented and the error bars represent the standard deviation (SD) for three individual measurements.

The kinetics of Trp fluorescence intensity changes of BSA and LYZ with polymers at fixed poly(B5AMA)/protein mass ratios of 1 and 8 were then studied for the first 6 h (Figure 3 and Figure S13). As shown in Figure 3, a decrease in the relative fluorescence signal ratio (the intensity at time t relative to that at t = 0) of BSA in the absence of any polymer was observed, and the addition of any polymer in a protein solution with a *w*/*w* ratio of 1 did not change the rate of decrease of the fluorescence ratio compared to BSA alone. In contrast, polymer–protein mixtures prepared at a *w*/*w* ratio of 8 slightly improved the fluorescence ratio for all stereoisomers of poly(B5AMA), implying weak interactions between the polymers and BSA at high *w*/*w* ratios. As expected [25], the PEG–protein system prepared at *w*/*w* ratios of 1 and 8 showed PEG concentration-dependent enhancement of protein fluorescence. Namely, negligible changes in protein fluorescence were observed at a *w*/*w* ratio of 1; however, at a PEG/protein ratio of 8, a rapid increase in Trp fluorescence was observed compared with protein alone, indicating stabilization of the protein in the presence of PEG at high *w*/*w* ratios.

Similarly, for LYZ, the kinetic fluorescent curves were essentially similar to those of proteins for PEG and for all stereoisomers of poly(B5AMA) at *w*/*w* ratios of 1 and 8, indicating no conformation changes in LYZ as a function of polymer concentration (Figure S13). The poly(B5AMA)-LYZ systems also showed a slower decrease in fluorescence ratio as a function of time when compared with BSA systems, indicating that the more flexible, high-molecular-weight BSA underwent relatively larger conformational changes compared with LYZ. These findings, in addition to the results shown in Figure 1 and Figure 2, indicate that BSA undergoes larger changes in the presence of stereoisomers of poly(B5AMA) at high *w*/*w* ratios; however, negligible polymer–protein interactions were observed for LYZ at all studied *w*/*w* ratios. As expected, the presence of PEG in the LYZ solution improved the overall stability of the protein, especially after the 4 h time point; however, unlike with BSA, the effect was independent of the mass ratio in the PEG–LYZ system.

The weak interactions of all stereoisomers of poly(B5AMA) with BSA, especially at low *w*/*w* ratios, and negligible interactions of the polymers with LYZ suggest the overall antifouling properties of the hydrophilic polymers. In prior studies, the antifouling properties of PEG and zwitterionic p(SBMA) upon interaction with LYZ and BSA in solution demonstrated no significant change in the fluorescence intensities of the model proteins after the addition of different mass ratios of hydrophilic and zwitterionic polymers in comparison with pure protein, suggesting negligible polymer/protein interactions at low *w*/*w* ratios [20,21,22,23,24,25,26,27]. Compared to non-ionic PEG, which is well-documented for its protein stabilization effects at high *w*/*w* ratios [20,21,22,23,24,25], p(SBMA) essentially showed no interactions with proteins at all studied *w*/*w* ratios [26,27,39,40]. 

### 3.2. ANS Fluorescence Characterization 

**Figure 4:**
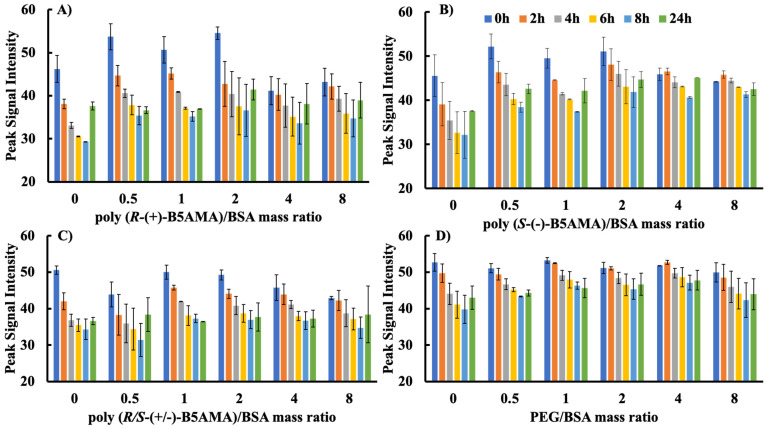
The characterization of polymer–protein interactions using ANS fluorescence dye upon changes in polymer/BSA (32 μg/mL) mass ratios (0, 0.5, 1, 2) for (**A**) poly(*R*-(+)-B5AMA_38_), (**B**) poly(*S*-(+)-B5AMA_38_), (**C**) poly(*R*/*S*-(+)-B5AMA_38_), and (**D**) PEG. The average values are presented and the error bars represent the standard deviation (SD) for three individual measurements. “0” represents pure BSA solution in the experiments.

ANS is a fluorescent probe that can quantitatively evaluate changes in the hydrophobic pockets of a protein. Specifically, the fluorescence intensity of ANS dye is greatly enhanced by its binding to the hydrophobic regions of a protein, which is a behaviour that can be used to demonstrate the conformational changes of proteins in the presence of polymers [25,26]. Because the fluorescence intensity of ANS shows a much larger polarity/environmental dependence than Trp, it provides a more sensitive fluorescence tool for studying changes in protein conformation and state. In order to compare polymer–BSA interactions, the fluorescence intensity of ANS was evaluated upon the incubation of BSA and ANS for 15 min prior to adding each polymer at different mass ratios (Figure 4). In the absence of polymers, the ANS fluorescence intensity of BSA gradually decreased with time, indicating the unfolding of the protein and a decrease in the availability of exposed hydrophobic pockets for ANS interaction, followed by an increase at 24 h, suggesting protein refolding. Upon addition of poly(*R*-(+)-B5AMA_38_) and poly(*S*-(+)-B5AMA_38_), the ANS fluorescence intensity of BSA slightly increased at time 0 h, possibly indicating slight interactions of pure isomers of poly(B5AMA) with BSA. The addition of poly(*R*/*S*-(+)-B5AMA_38_) and PEG, however, did not significantly change the ANS fluorescence intensity of the BSA solution. The ANS fluorescence intensity of BSA in the presence of all polymers studied also decreased with time (over a period of 8 h); however the decrease in fluorescence intensity was slower in the presence of PEG, poly(*R*-(+)-B5AMA_38_), and poly(*S*-(+)-B5AMA_38_) at polymer/BSA *w*/*w* ratios of 0.5, 1, and 2 compared to the ANS fluorescence intensity of a pure protein solution. Interestingly, in the presence of poly(*R*/*S*-(+)-B5AMA_38_), the ANS fluorescence intensity of BSA remained unchanged in the presence and absence of polymers. It should be noted that there were only slight changes (5–7 nm) in the emission maxima of ANS dye in the presence of different mass ratios of polymers to proteins (Figures S14–S17).

Rawat [23] and Wu et al. [25] previously reported that the addition of PEG long chains (>5 kDa) in BSA–ANS systems leads to higher ANS signal intensity, indicating that PEG chains stabilize the protein structure and maintain hydrophobic cavities for ANS binding. PEG–BSA interactions are mainly attributed to van der Waals forces, and the open structure of the protein can provide more hydrophobic sites for PEG to interact. Looking at the ANS and Trp fluorescence results together, poly(*R*-(+)-B5AMA_38_) and poly(*S*-(+)-B5AMA_38_) showed similar behaviour to PEG and showed polymer concentration-dependent interactions with the proteins, possibly by weak hydrophilic and van der Waals forces. Poly(B5AMA)-BSA systems prepared at different *w*/*w* ratios were further studied by CD and NMR spectroscopy to evaluate the changes in the secondary structure of the proteins in the presence of hydrophilic polymers. 

### 3.3. CD Spectroscopy

**Figure 5:**
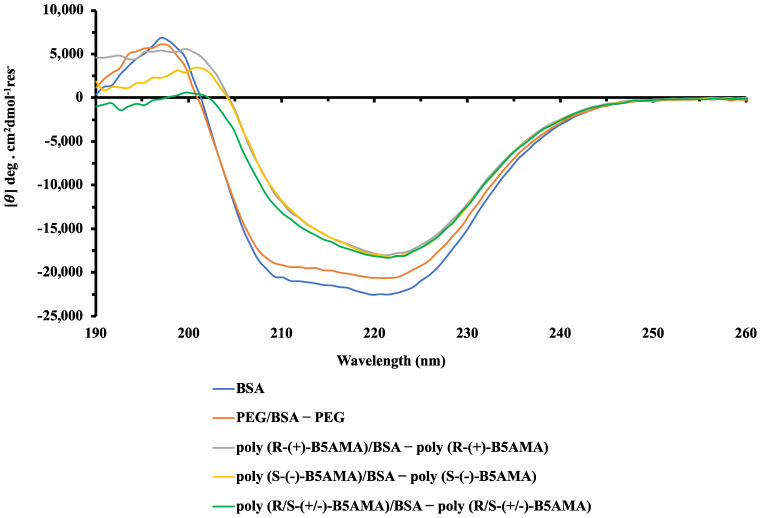
CD spectra indicating the interactions between indicated polymer–BSA mixtures prepared at a *w*/*w* ratio of 1. The spectrum of BSA alone is overlaid with different spectra representative of BSA conformation in a given condition derived by the subtraction of the CD spectrum of polymer alone from that of the polymer–BSA mixture.

Far-UV CD analysis was performed in a PBS buffer at polymer/protein *w*/*w* ratios of 1 and 8. The CD spectrum of free BSA and the CD difference spectra of polymer–BSA mixtures prepared at a *w*/*w* ratio of 1 demonstrated similar line shapes as those for free BSA and PEG–BSA mixtures (Figure 5). In each case, negative bands were clear at 208 and 222 nm with a positive band at ~195 nm, a characteristic pattern for α-helix-rich proteins [41], with a slight reduction in intensity in the presence of PEG being consistent with a slight reduction in the α-helicity of BSA. Contrary to this, the addition of each of the three stereoisomers of poly(B5AMA) appeared to lead to a greater loss of α-helical character in BSA, apparent from the decrease in CD intensity that was observed in the 210–225 nm region of the spectrum. As discussed above, due to the presence of chain transfer end groups, poly(B5AMA) stereoisomers absorb relatively strongly below ~210 nm and interfere with the ability to observe α-helical bands at 208 nm and 195 nm. The apparent disappearance of these bands in the difference spectra is, therefore, not taken to be indicative of a complete loss of α-helicity. It should also be noted that this spectral interference also makes CD deconvolution infeasible. In summary, the observed changes in the CD spectral pattern for BSA were highly consistent for poly(*R*-(+)-B5AMA_38_), poly(*S*-(+)-B5AMA_38_), and poly(R/*S*-(+/−)-B5AMA_38_). CD spectra of poly(B5AMA)–BSA mixtures prepared at high *w*/*w* ratios were not obtained as polymer absorbance in the UV region interfered with data acquisition and analysis.

### 3.4. Thermal Denaturation

**Figure 6:**
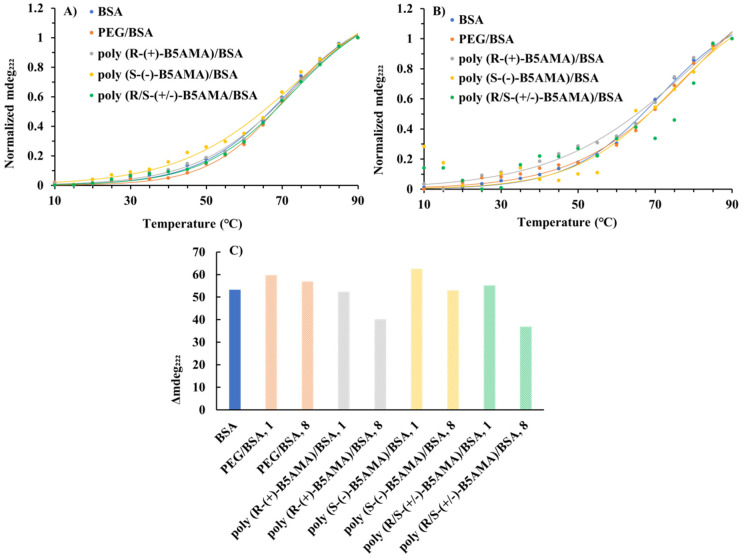
Thermal denaturation study of BSA in the presence of different polymers. Normalized CD signal at 222 nm observed as a function of temperature during thermal denaturation of BSA for polymer/BSA *w*/*w* ratios of 1 (**A**) and 8 (**B**). Lines of fit using the logistic sigmoidal model (Equation (1)) are shown for all datasets except poly(*R*/*S*-(+/−)B5AMA)/BSA prepared at a *w*/*w* ratio of 8 due to poor reliability of that fit. The change in ellipticity at 222 nm between the minimum (low temperature) and maximum (high temperature) observed for the indicated sample during thermal denaturation was monitored by CD spectroscopy (**C**). Each sample was prepared in PBS at pH 7.4 with a final concentration of 0.5 mg/mL BSA. Normalization is presented with respect to extremes for a given sample type, not with respect to global extremes in ellipticity.

The interactions of polymers with BSA at different *w*/*w* ratios (1 and 8) were further analyzed by measuring the thermal denaturation profile of BSA in the presence and absence of the polymers using CD spectroscopy at 222 nm, where the CD signal was relatively unaffected by the polymers. The thermal denaturation profiles of polymer–BSA mixtures at *w*/*w* ratios of 1 and 8 for each polymer were normalized and compared with that of BSA alone. To ensure that the polymers alone were not contributing to the observed denaturation profiles, CD measurements of each polymer were also carried out (Figure S18). These control experiments remained similar throughout the temperature range employed, suggesting that any changes observed were due to the interactions between BSA and polymers and not due to a polymer-related CD spectral feature. All polymer–BSA mixtures prepared at a *w*/*w* ratio of 1 exhibited similar thermal denaturation profiles to that of BSA alone, with BSA being largely denatured at the assay end-point of 90 °C (Figure 6A) and with T_m_ values for each sample of ~71–73 °C. All mixtures of BSA prepared with stereoisomers poly(B5AMA) at a *w*/*w* ratio of 1 demonstrated modest thermal stabilization relative to either BSA alone or BSA-PEG mixture (Table 2). This was consistent with the observed interactions of each of the forms of poly(B5AMA) with BSA at a *w*/*w* ratio of 1 by far-UV CD spectroscopy (Figure 6).

The addition of the polymers at a polymer/BSA *w*/*w* ratio of 8 appeared to enhance the thermal stability of BSA to varying degrees from ~74 to 80 °C (Figure 6B and Table 2). Evaluating this in more depth, the difference between the minimum and maximum ellipticity values (Δ_222_) observed is indicative of the relative degree of structural perturbation induced in each instance, where each poly(B5AMA) stereoisomer–BSA mixture prepared at a *w*/*w* ratio of 8 exhibited a lesser Δ_222_ in comparison to the same polymer–BSA mixtures prepared at a *w*/*w* ratio of 1 and a lesser Δ_222_ compared to either the PEG–BSA mixtures or BSA alone (Figure 6C). This is consistent with greater thermal stabilization for the poly(*R*-(+)-B5AMA_38_), poly(*S*-(−)-B5AMA_38_), and poly(*R*/*S*-(+/−)-B5AMA_38_) protein mixtures at *w*/*w* ratios of 8, which appear not to have fully reached the denaturation plateau at 90 °C, despite good fits being achieved for the thermal denaturation in the presence of poly(*R*-(+)-B5AMA_38_) and poly(*S*-(−)-B5AMA_38_). This behaviour suggests that poly(B5AMA) stereoisomers at high concentrations all behave distinctly from PEG in acting as stabilizing agents for BSA.

Messina et al. demonstrated that the stereoisomers of poly(trehalose) show limited interactions and no stabilization effect of proteins at a polymer/protein *w*/*w* ratio of 1, whereas an enhanced stabilization effect was observed at a polymer/protein *w*/*w* ratio of 10 under mechanical stress [6]. We have also previously demonstrated that poly(B5AMA)-based hydrogels act as macromolecular chaperones and stabilize the tertiary structure of LYZ at elevated temperatures [31]. The chaperone-like activities of these hydrogels were attributed to the hydration capability of hydrogels as a function of temperature providing protein stabilization via hydrogen bonding and hydrophobic interactions [31]. The enhanced thermal stability of BSA in the presence of stereoisomers of poly(B5AMA) further validates that the hydration potential of these polymers is the key factor in protein stability.

### 3.5. NMR Spectroscopy

**Figure 7:**
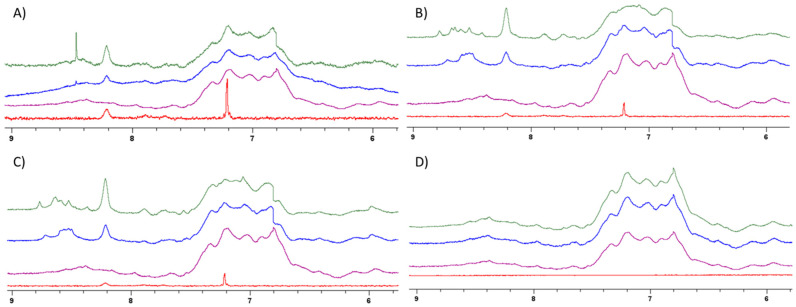
Titration of BSA in D_2_O ^1^H NMR signals upon incubation with (**A**) poly (*R*-(+)-B5AMA_38_), (**B**) poly(*S*-(−)-B5AMA_38_), (**C**) poly(*R*/*S*-(+/−)-B5AMA_38_), and (**D**) PEG; in order from bottom to top are polymer alone (red), protein alone (purple), and polymer/protein *w*/*w* ratio of 1 (blue) and 2 (green).

A 1D ^1^H NMR study was then conducted to investigate the interaction between BSA and polymers, as NMR spectroscopy has previously been used to study interactions between and assign binding sites in polymers and proteins [26]. To do this, PEG or poly(B5AMA) was incubated with BSA at specific polymer/protein ratios of 0, 1, and 2 (Figure 7). Figure 7 shows the region of the ^1^H NMR spectrum containing the signal corresponding to backbone amide, side-chain amide, and side-chain aromatic resonances—including those of Trp [26]. As can be seen in Figure 7, no significant change in chemical shift or peak pattern was observed for the poly(*R*-(+)-B5AMA_38_) and PEG samples at all *w*/*w* ratios studied. However, there was a slight variation in peak shape observed for the poly(*S*-(−)-B5AMA_38_) and poly(*R*/*S*-(+/−)-B5AMA_38_) samples, especially at a polymer/protein *w*/*w* ratio of 2, suggesting the potential for interaction between BSA and the optically active polymer, especially at high *w*/*w* ratios. Studies at *w*/*w* ratios greater than 2 were uninterpretable as the high polymer content of these samples obscured the protein signals. The slight variation in signal intensity in NMR spectra of all samples is attributed to variations in concentrations of the polymer and protein.

## 4. Conclusions and Future Directions

In this study, we investigated the nonspecific physical interactions of stereoisomers of poly(B5AMA) and PEG with the model proteins BSA and LYZ utilizing steady-state Trp fluorescence spectroscopy, ANS fluorescence, CD spectroscopy, and ^1^H NMR spectroscopy in an aqueous solution. Taken together, fluorescence spectroscopy revealed that hydrophilic poly(B5AMA) and PEG exhibit concentration-dependent protein stabilizing effects, as was observed at different *w*/*w* ratios of polymers to proteins. Polymer–BSA systems prepared at low *w*/*w* ratios showed no significant change in the fluorescence intensity of Trp residues of BSA. However, at high *w*/*w* ratios, a stabilizing effect of PEG was clear, with increased Trp fluorescence intensity. Poly(B5AMA) stereoisomers added to BSA at high *w*/*w* ratios, in contrast, showed a decrease in the fluorescence intensity of Trp, a quenching phenomenon that may be attributable to the photophysical interaction between Trp and the CTA region of the polymer. Far-UV CD spectroscopy and thermal denaturation of the protein followed by CD spectroscopy were employed to further explore polymer–BSA interactions. Although CD spectroscopy showed strong interference of the polymers in far-UV regions of the spectra, the thermal denaturation profiles indicated that poly(B5AMA)–protein systems prepared at a *w*/*w* ratio of 1 did not impact the protein denaturation profile; however, at high *w*/*w* ratios, all polymers showed significant protein stabilizing abilities. Interestingly, the stabilizing effect of BSA was significantly different for PEG in comparison with poly(B5AMA). ^1^H NMR studies were then performed to further evaluate the potential for changes in the secondary and tertiary structures of both BSA and LYZ in the presence of the polymers. NMR analysis implied no significant changes in the backbone amide, side-chain amide, or aromatic residues of either protein in the presence of PEG or poly(*R*-(+)-B5AMA_38_); however, a broadening of peaks was observed in the presence of high *w*/*w* ratios of poly(*S*-(−)-B5AMA_38_) and poly(*R*/*S*-(+/−)-B5AMA_38_), which may indicate variable interactions of optically active polymers with the proteins.

In summary, the development of novel telechelic stealth polymers by a living radical polymerization approach has been the focus of intense research over the last two decades. Stealth polymers prepared by living methods may incorporate functional groups that interfere with the study of their interactions with proteins in solution. The use of multiple biophysical analysis techniques may thus be required to decipher the interactions of polymers with proteins in detail. Future work will focus on the development of CTA hydrolyzed poly(B5AMA) and the evaluation of their interactions with proteins as a function of end-group functionalities. Furthermore, our NMR analysis indicates the role of the optical activity of poly(B5AMA) in protein interactions at high *w*/*w* ratios of polymers. Optically active poly(B5AMA) of different molecular weights will be prepared to further understand the role of polymer size in protein interactions and stability.

## Figures and Tables

**Table 1:** Molecular weight (number average (M_n_) and weight average (M_w_)), polydispersity index (PDI), and optical activity of stereoisomers of poly(B5AMA) and PEG control.

Sample	M_n_ (g/mol)	M_w_ (g/mol)	PDI	Optical Activity
Poly (*R*-(+)-B5AMA)_38_	7942	8943	1.13	+30.9°
Poly (*S*-(+)-B5AMA)_38_	7777	8877	1.14	−34.8°
Poly (*R*/*S*-(+/−)-B5AMA)_38_	7754	8867	1.14	−4.8°
PEG	10,000	-	n/a	0

**Table 2:** Thermal denaturation midpoint (T_m_) for given sample based upon CD spectroscopy signal at 222 nm using a fit of the logistic sigmoidal model (Equation (1)) to the data.

Sample	T_m_ (°C) [Polymer:BSA Ratio]	
BSA	71	
PEG/BSA	71 [1]	79 [8]
Poly (*R*-(+)-B5AMA)_38_/BSA	72 [1]	80 [8]
Poly (*S*-(−)-B5AMA)_38_/BSA	73 [1]	74 [8]
Poly (R/*S*-(+/−)-B5AMA)_38_/BSA	72 [1]	N/D ^a^

^a^ Not determined (N/D) due to poor reliability of fit.

## Data Availability

The data presented in this study are available in the main article and Supplementary Materials.

## Supplementary Materials

The following supporting information can be downloaded at: https://www.mdpi.com/article/10.3390/polym16010065/s1.
