# Supplementary material for: Elucidating the Role of Optical Activity of Polymers in Protein–Polymer Interactions
Source: Polymers (Basel). 2023 Dec 24;16(1):65. doi: 10.3390/polym16010065 (PMC10781056; doi:10.3390/polym16010065)
Supplement: Supplementary file 1 [file polymers-16-00065-s001.zip › polymers-2753978-supplementary.pdf]

Elucidating the Role of Optical Activity of Polymers in Protein-Polymer Interactions

Samin Jahan[1], Catherine Doyle[1], Diego Combita[1], Anupama Ghimire[2], Jan K. Rainey[2-4], Brian D. Wagner,[1] Marya Ahmed[1,5*]

Department of Chemistry University of Prince Edward Island, Charlottetown, Prince Edward Island, Canada, C1A 4P3[1], Department of Biochemistry & Molecular Biology[2], Department of Chemistry[3], School of Biomedical Engineering, Dalhousie University, Halifax, Nova Scotia B3H 4R2, Canada[4]; Faculty of Sustainable Design Engineering[5]; University of Prince Edward Island, Charlottetown, Prince Edward Island, Canada, C1A 4P3

Corresponding email address: marahmed@upei.ca

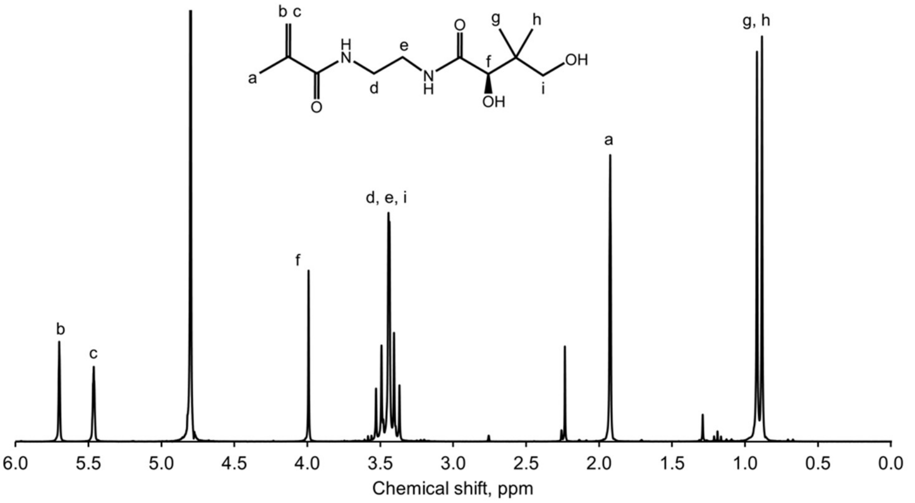

Figure S1: ^{1}H NMR spectrum of *R*-(+)-B5AMA monomer.

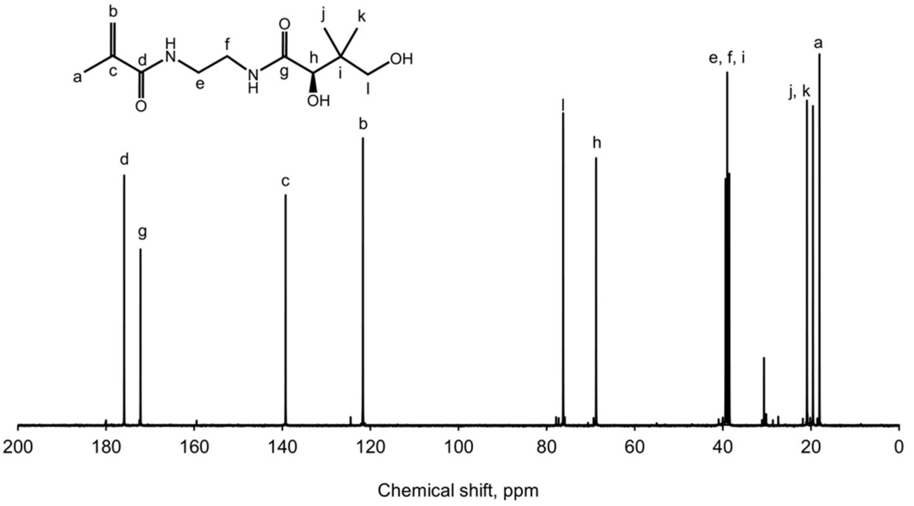

Figure S2: ^{13}C NMR spectrum of *R*-(+)-B5AMA monomer.

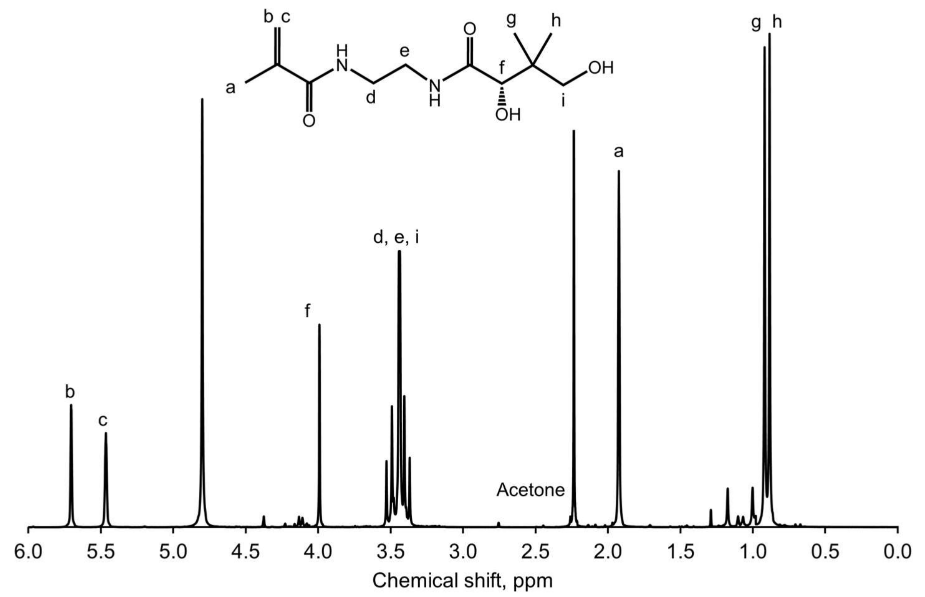

Figure S3: ^{1}H NMR spectrum of *S*-(-)-B5AMA monomer.

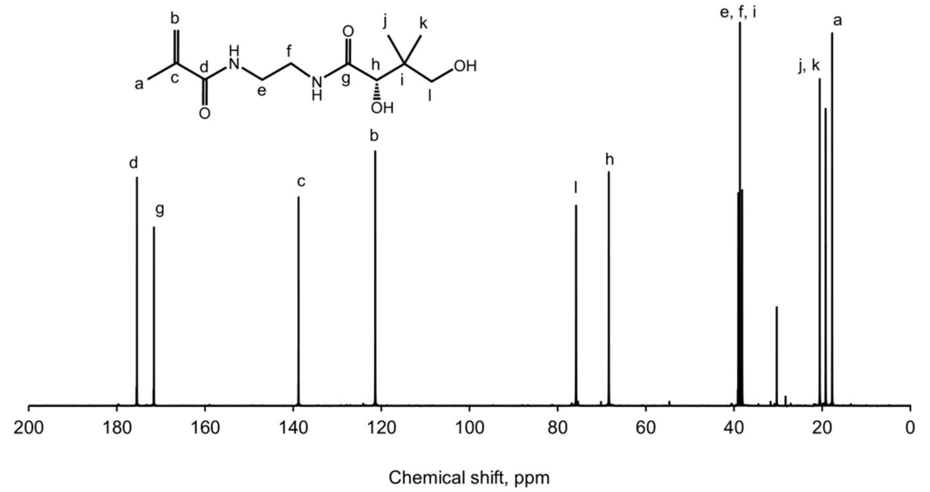

Figure S4: ^{13}C NMR spectrum of *S*-(-)-B5AMA monomer.

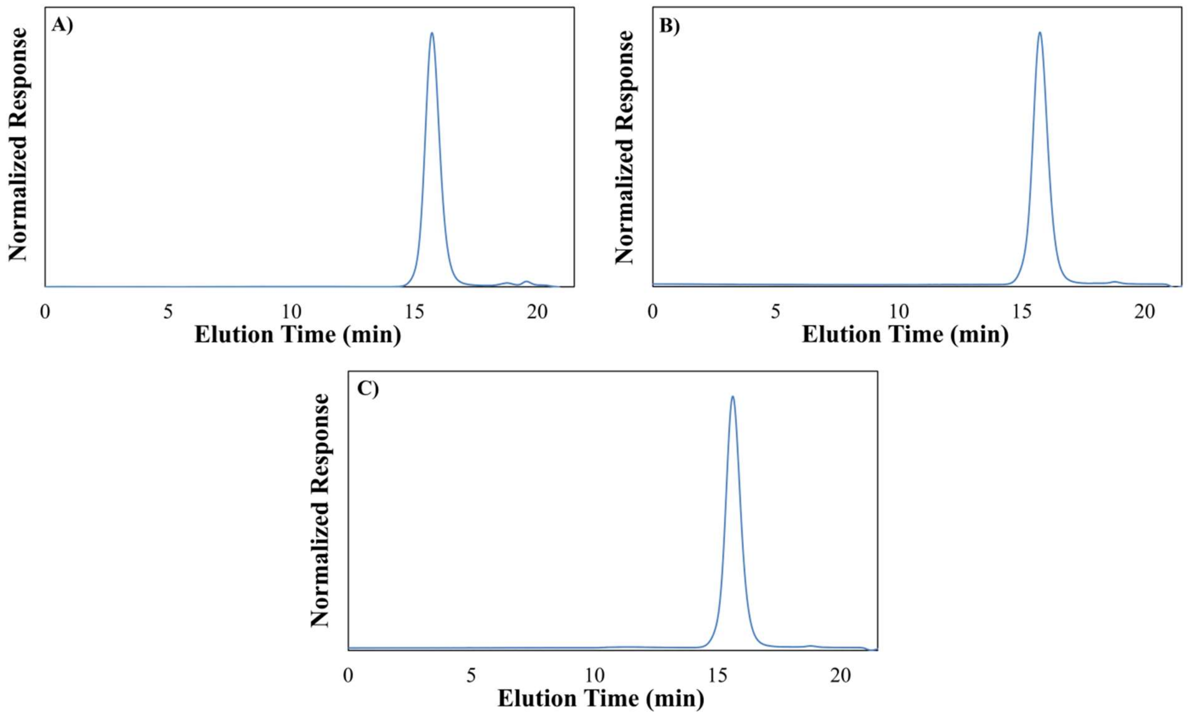

Figure S5: GPC Traces of poly(*R*-(+)-B5AMA$_{38}$) A), poly(*S*-(-)-B5AMA$_{38}$) B), and poly(*R*/*S*-(+/-)-B5AMA$_{38}$) C).

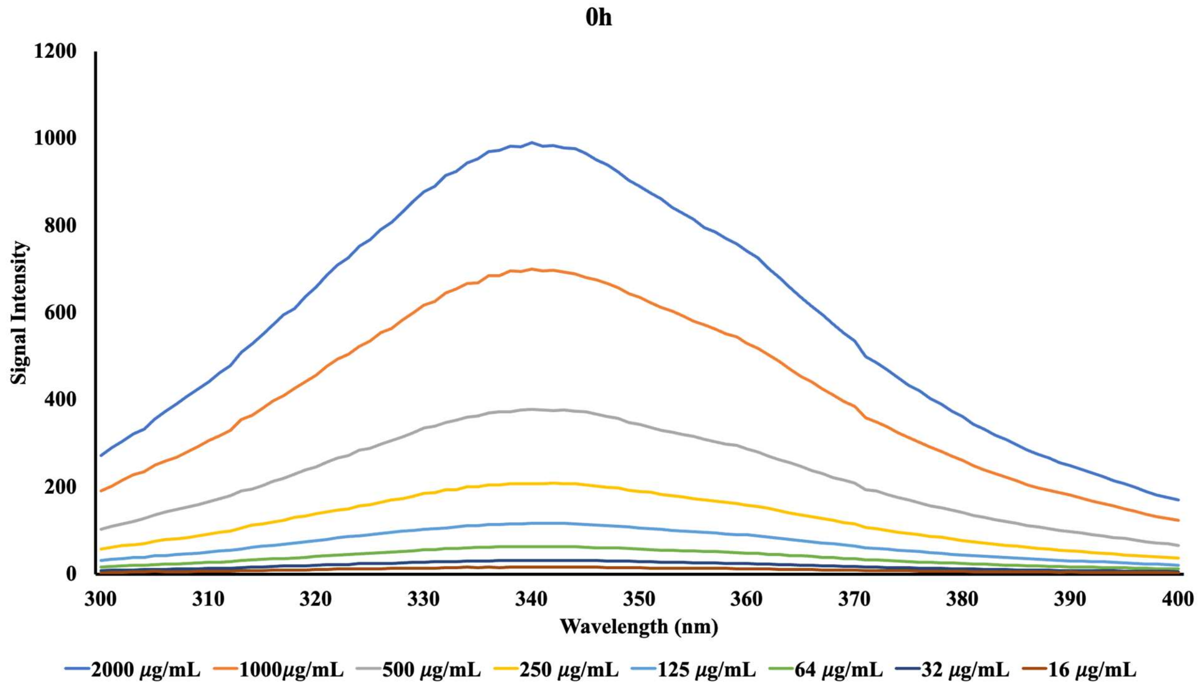

Figure S6: Fluorescence intensity of BSA Tryptophan residue as a function of concentration of protein at 0h. Excitation wavelength is 279 nm and emission spectra are recorded from 300-400 nm for BSA.

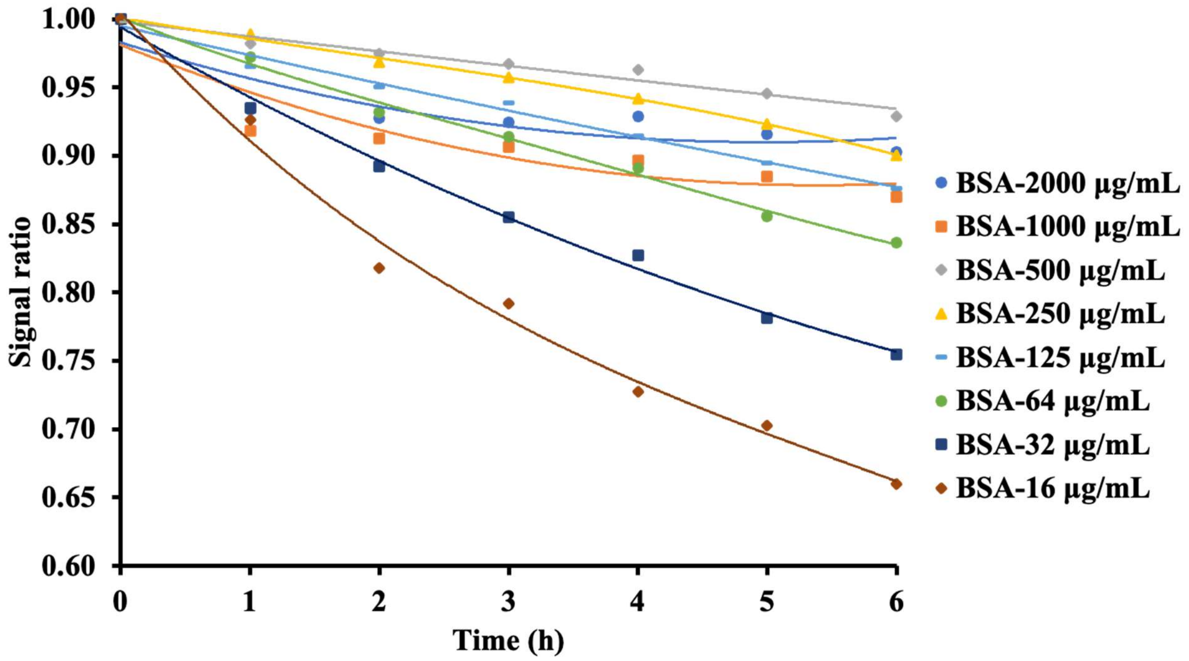

Figure S7: Normalized fluorescence signal of Trp residue of BSA at different concentrations of the protein.

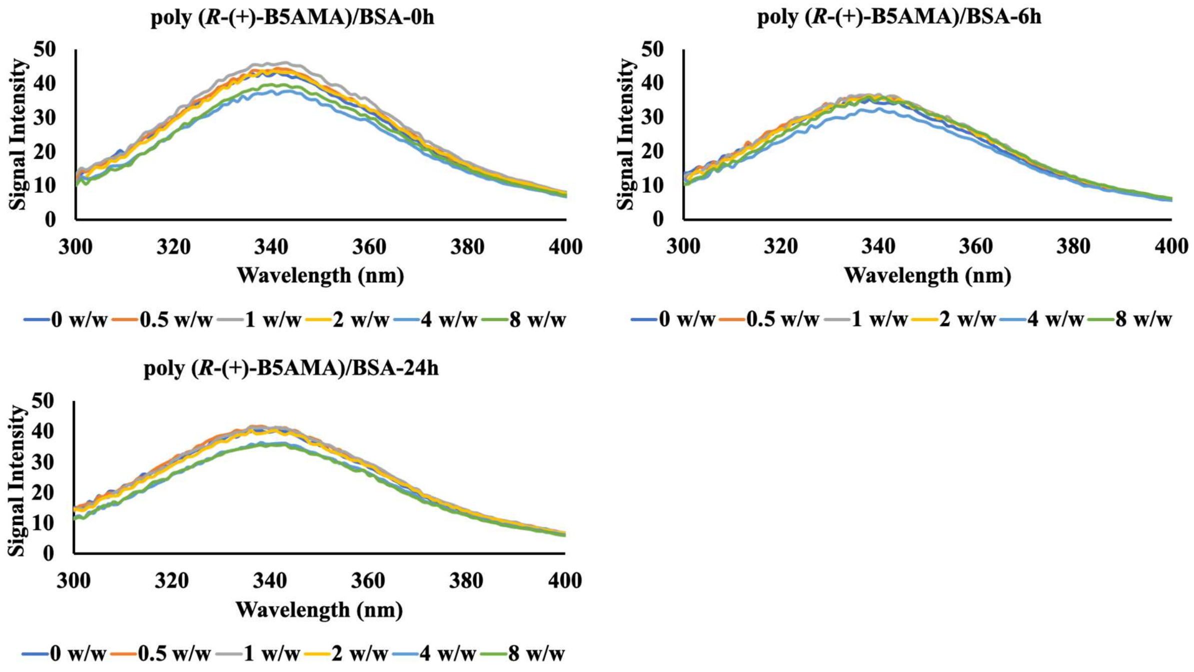

Figure S8: Fluorescence intensity of Trp residue of BSA, at different w/w ratios of poly (*R*-(+)-B5AMA)/BSA. The fluorescence spectra is obtained at excitation wavelength of 279 nm and emission wavelength is 300-400nm at different time points.

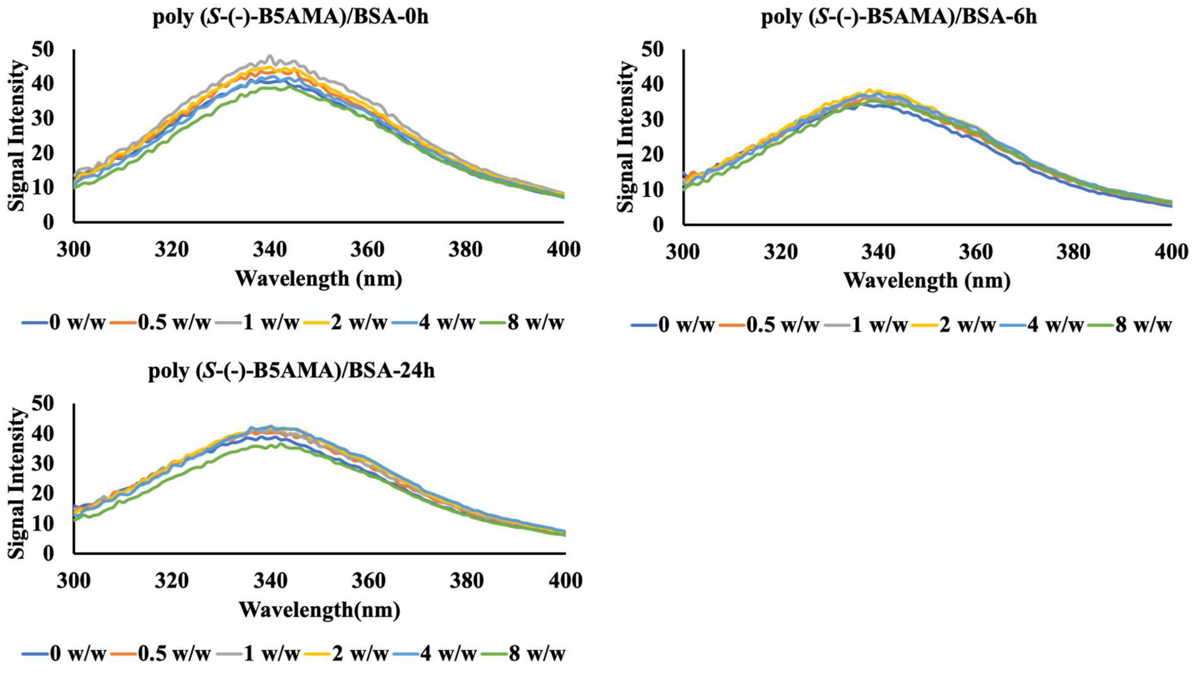

Figure S9: Fluorescence intensity of Trp residue of BSA, at different w/w ratios of poly (*S*-(-)-B5AMA)/BSA. The fluorescence spectra is obtained at excitation wavelength of 279 nm and emission wavelength is 300-400nm at different time points.

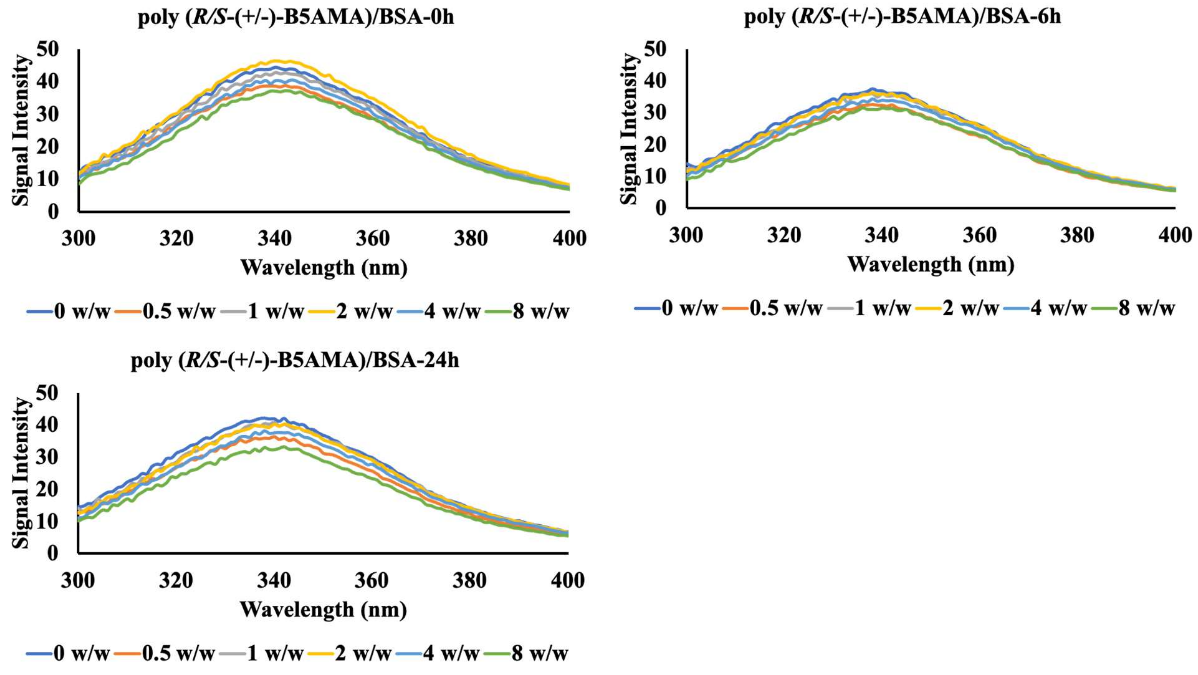

Figure S10: Fluorescence intensity of Trp residue of BSA, at different w/w ratios of poly (R/S-(+/-)-B5AMA)/BSA. The fluorescence spectra is obtained at excitation wavelength of 279 nm and emission wavelength is 300-400nm at different time points.

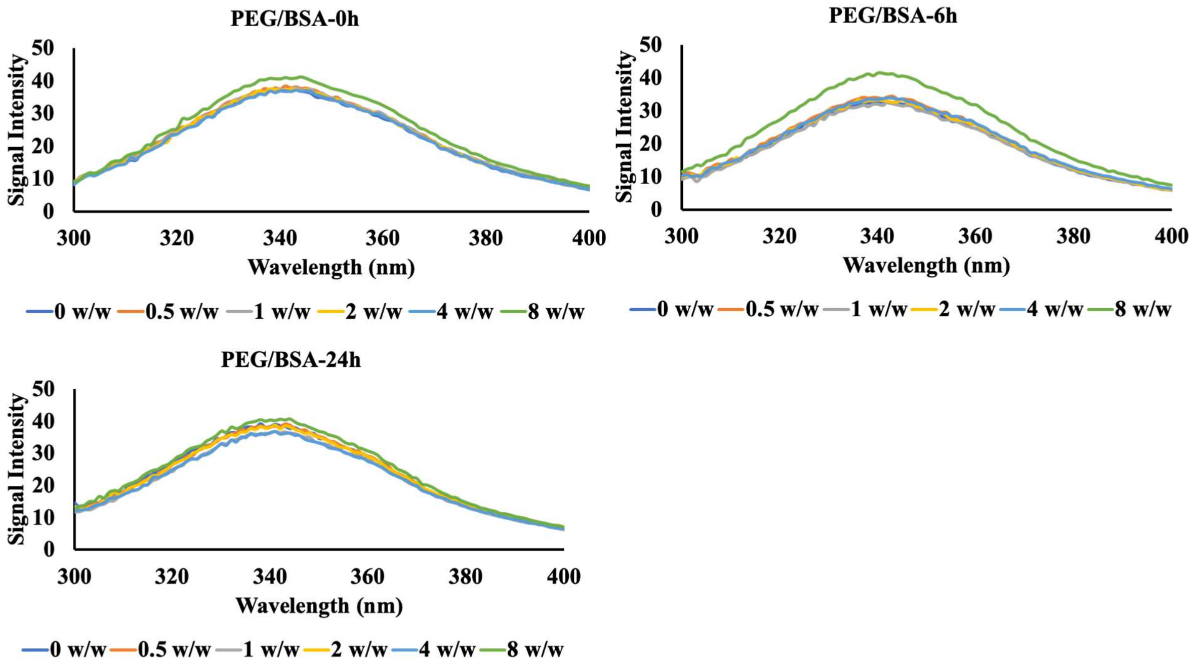

Figure S11: Fluorescence intensity of Trp residue of BSA, at different w/w ratios of PEG/BSA. The fluorescence spectra is obtained at excitation wavelength of 279 nm and emission wavelength is 300-400nm at different time points.

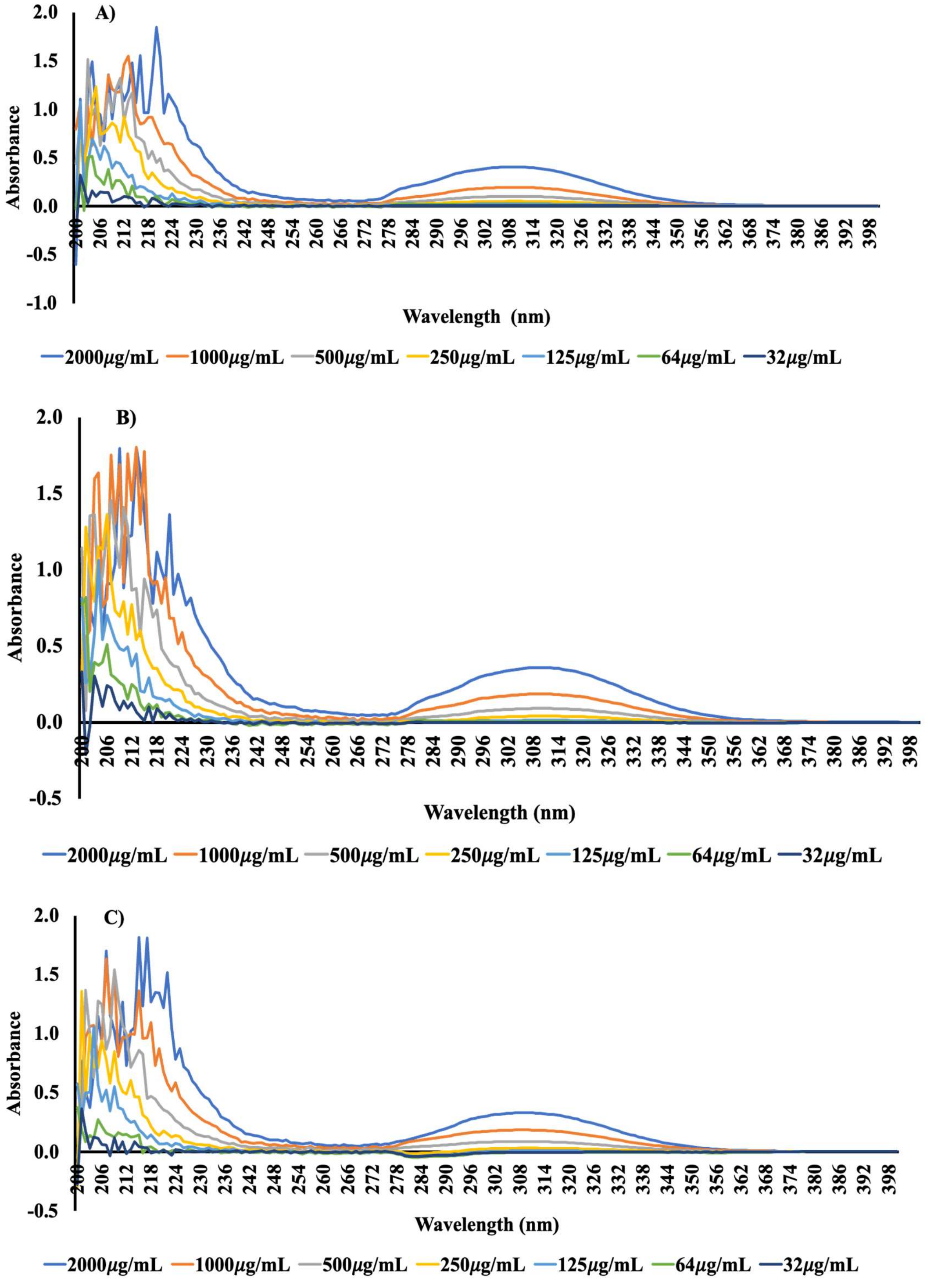

Figure S12: Poly (*R*-(+)-B5AMA) A), poly (*S*-(-)-B5AMA) B), and poly (*R/S*-(+/-)-B5AMA) C) absorbance spectrum from 200 to 400nm.

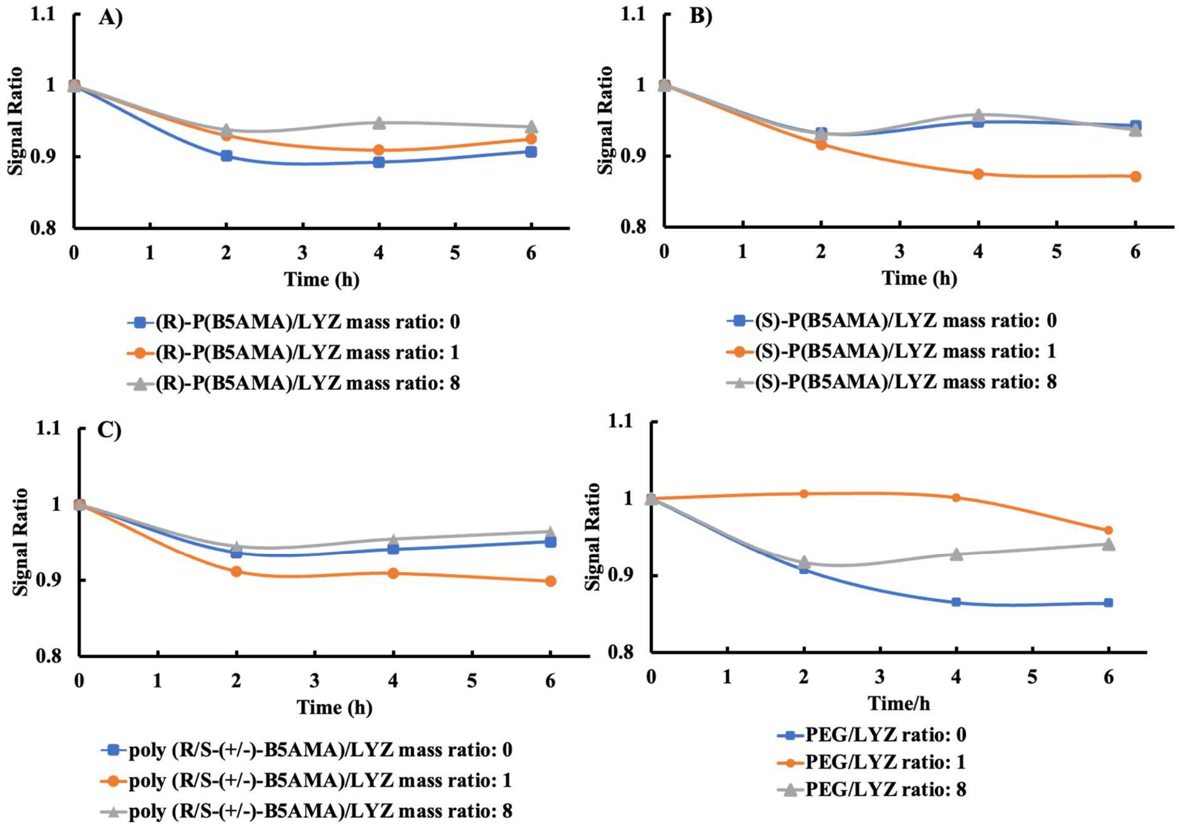

Figure S13: Normalized fluorescence intensity of LYZ with and without poly (*R*-(+)-B5AMA) A), poly (*S*-(-)-B5AMA) B), poly (*R/S*-(+/-)-B5AMA) C), and PEG D). Normalized fluorescence intensity is determined at polymer: protein ratio of 1:1 and 1:8 and at different time points.

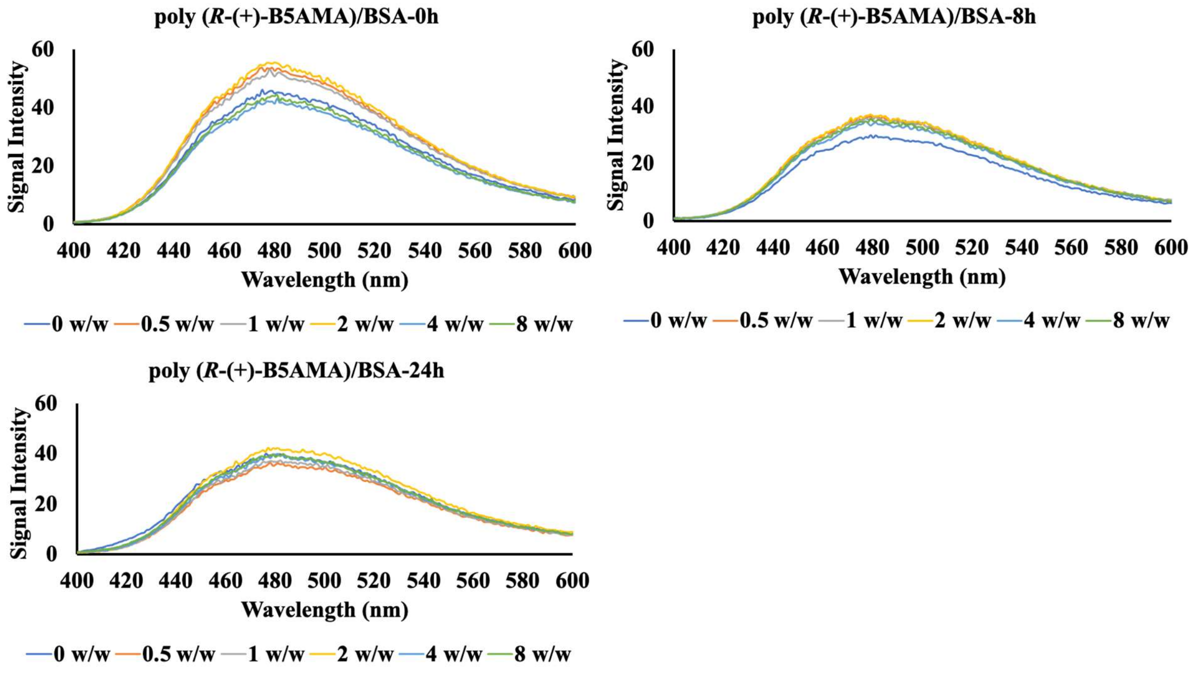

Figure S14: Fluorescence intensity of ANS upon interaction with BSA, at different w/w ratios of poly (*R*-(+)-B5AMA)/BSA. The fluorescence spectra is obtained at excitation wavelength of 279 nm and emission wavelength is 300-400nm at different time points.

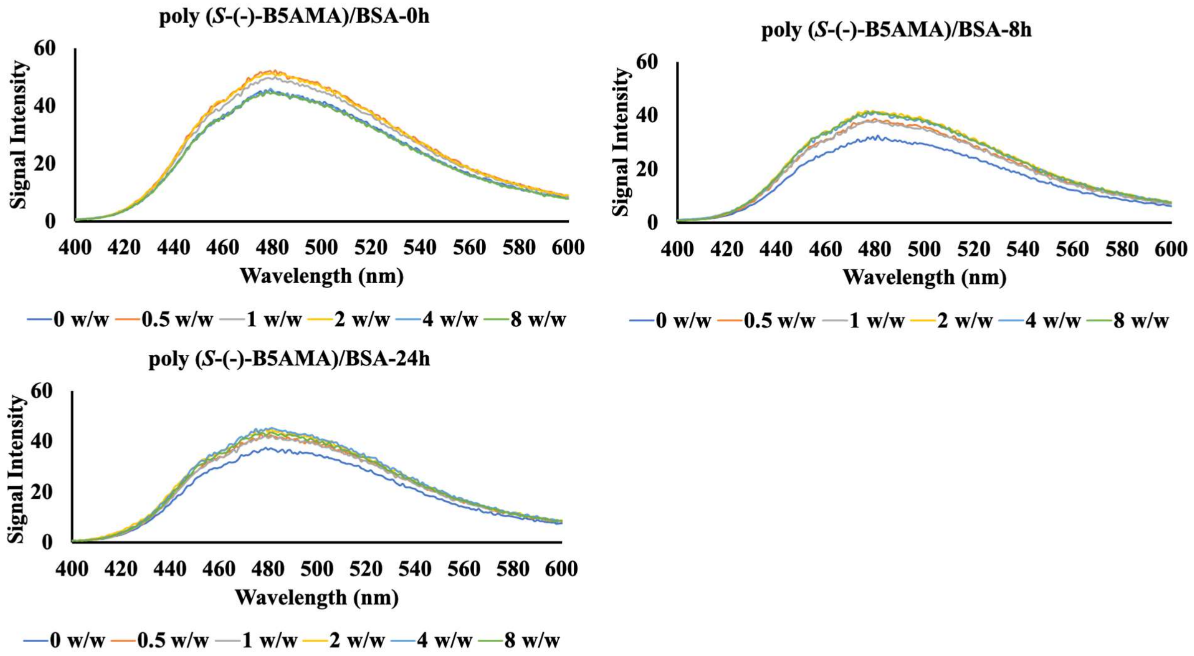

Figure S15: Fluorescence intensity of ANS upon interaction with BSA, at different w/w ratios of poly (*S*-(-)-B5AMA)/BSA. The fluorescence spectra is obtained at excitation wavelength of 279 nm and emission wavelength is 300-400nm at different time points.

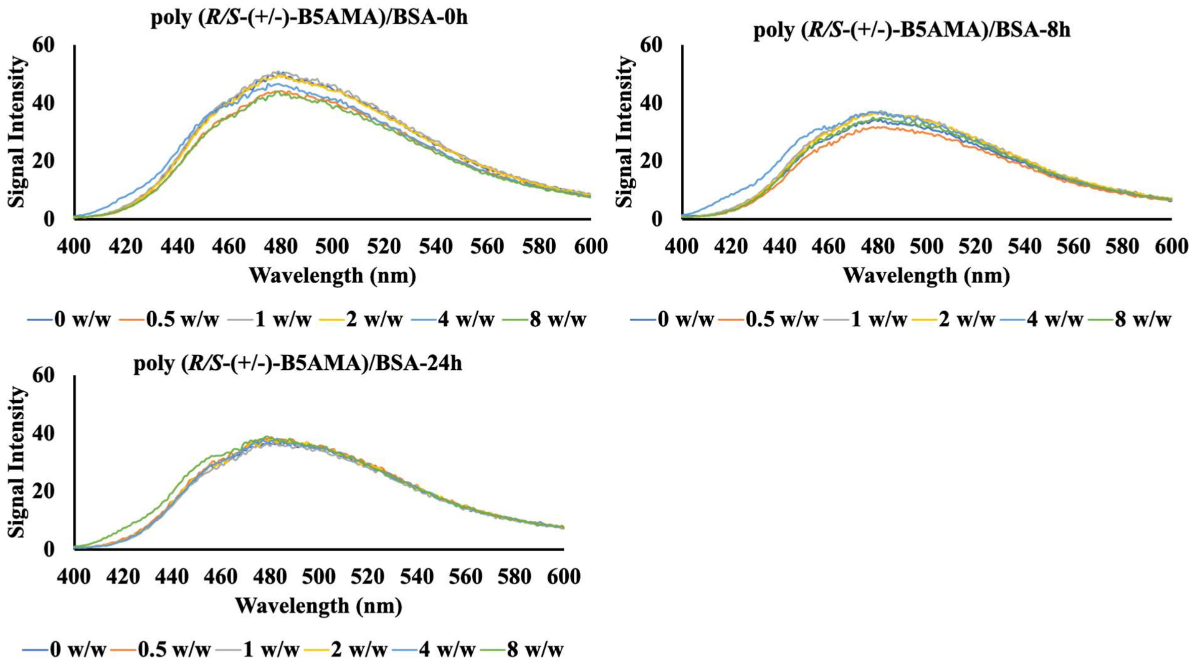

Figure S16: Fluorescence intensity of ANS upon interaction with BSA, at different w/w ratios of poly (*R/S*-(+/-)-B5AMA)/BSA. The fluorescence spectra is obtained at excitation wavelength of 279 nm and emission wavelength is 300-400nm at different time points.

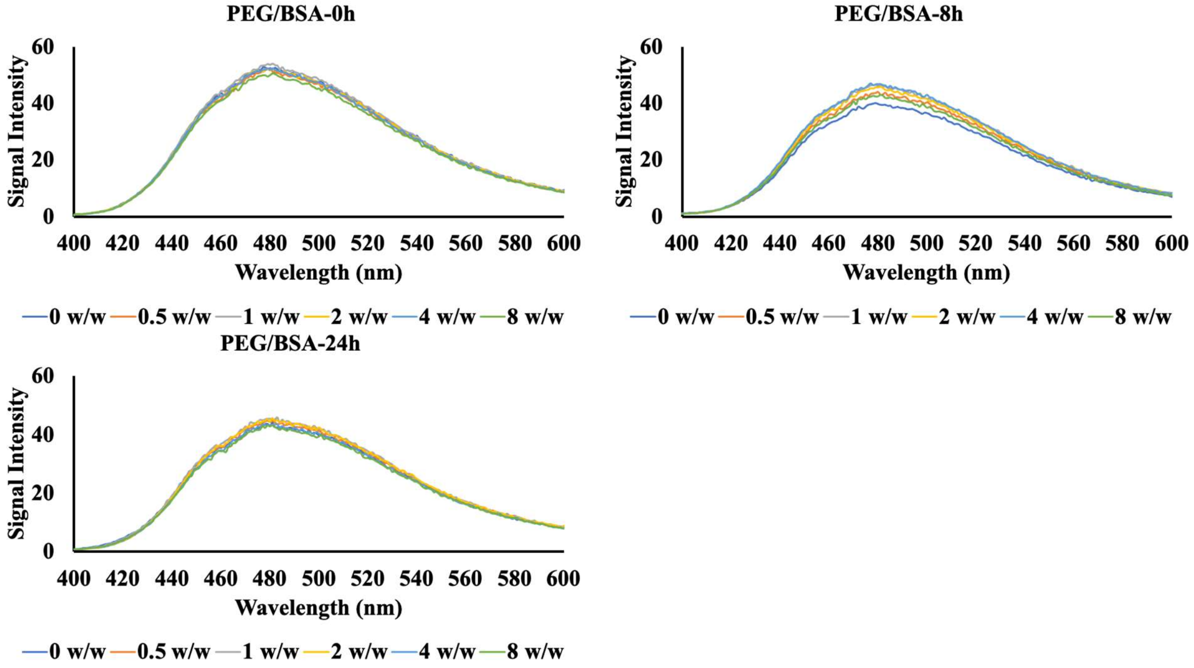

Figure S17: Fluorescence intensity of ANS upon interaction with BSA, at different w/w ratios of PEG/BSA. The fluorescence spectra is obtained at excitation wavelength of 279 nm and emission wavelength is 300-400nm at different time points.

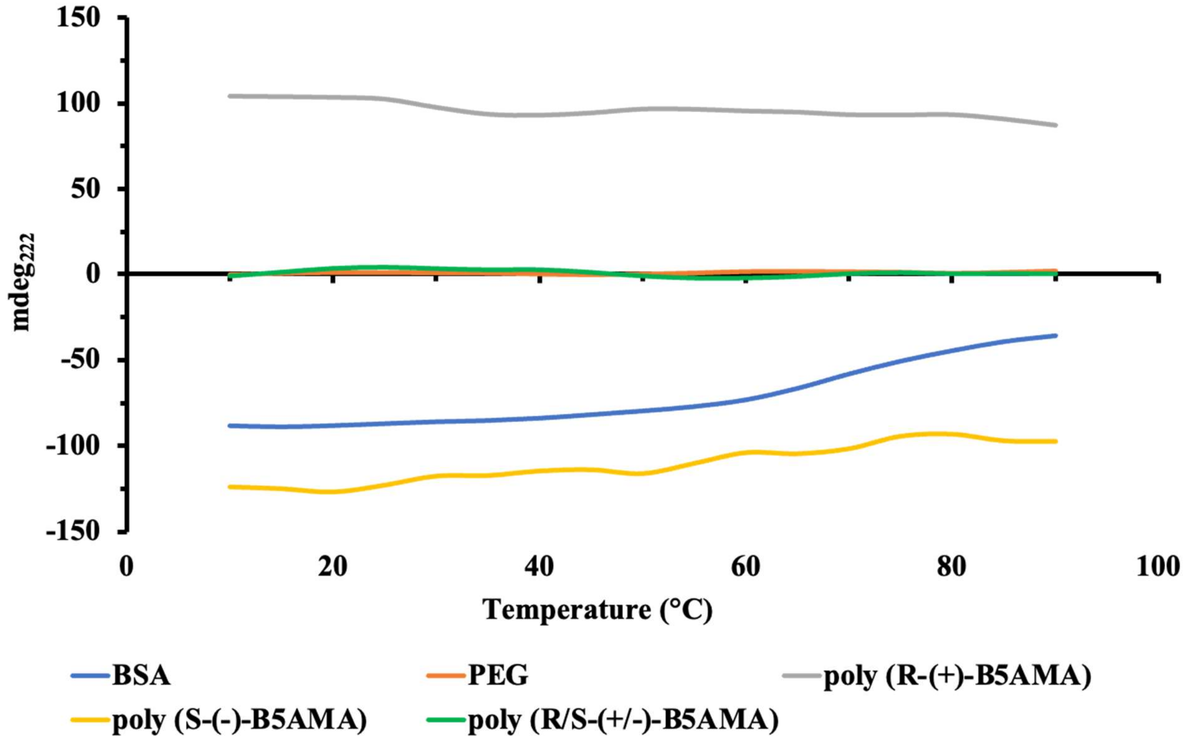

Figure S18: CD measurements at 222 nm during thermal denaturation of each polymer with a final concentration of 4 mg/mL.
